# Vaccine Interactions With the Infant Microbiome: Do They Define Health and Disease?

**DOI:** 10.3389/fped.2020.565368

**Published:** 2020-11-26

**Authors:** Candice E. Ruck, Oludare A. Odumade, Kinga K. Smolen

**Affiliations:** ^1^Department of Experimental Medicine, University of British Columbia, Vancouver, BC, Canada; ^2^Precision Vaccines Program, Division of Infectious Diseases, Boston Children's Hospital, Boston, MA, United States; ^3^Harvard Medical School, Boston, MA, United States; ^4^Division of Medicine Critical Care, Boston Children's Hospital, Harvard Medical School, Boston, MA, United States; ^5^Institute for Medical Immunology, Université libre de Bruxelles, Brussels, Belgium

**Keywords:** microbiome, vaccines, infant microbiome, immune system, maternal microbiome

## Abstract

Over the past decade, there has been a growing awareness of the vital role of the microbiome in the function of the immune system. Recently, several studies have demonstrated a relationship between the composition of the microbiome and the vaccine-specific immune response. As a result of these findings, the administration of probiotics has been proposed as a means of boosting vaccine-specific immunity. Early results have so far been highly inconsistent, with little evidence of sustained benefit. To date, a precise determination of the aspects of the microbiome that impact immunity is still lacking, and the mechanisms of action are also unknown. Further investigations into these questions are necessary to effectively manipulate the microbiome for the purpose of boosting immunity and enhancing vaccine-specific responses in infants. In this review, we summarize recent studies aimed at altering the neonatal gut microbiome to enhance vaccine responses and highlight gaps in knowledge and understanding. We also discuss research strategies aimed at filling these gaps and developing potential therapeutic interventions.

## Introduction

Following the relative sterility of the intrauterine environment, the microbiome is seeded from the earliest postnatal microbial exposures. The early life maturation of the microbiome also coincides with the period when the immune system is undergoing rapid development and is most susceptible to perturbations in the microbial environment. This early period of microbial and immunological maturation coincides with the timing of the primary series of immunizations. Thus, these early influences have the potential to have significant long-term effects on the development of vaccine-specific immunity.

## The Evolution of the Infant Microbiome

Bacterial diversity increases over the first year of life (1) and by 2 years of age much of the individual variability of the infant microbiota has converged into a relatively stable profile that resembles that of the typical adult (2–4). The dominant phyla in the intestinal microbiome of a healthy adult are Firmicutes and Bacteroidetes, while in the infant gut, members of the phylum Actinobacteria often predominate (5). During this early period of rapid intestinal colonization, the relative abundance and diversity within each phyla may be influenced by a multitude of factors. Principal among these are mode of delivery, mode of feeding, environmental, and genetics (3).

### Mode of Delivery

The long-standing view that the *in utero* environment is sterile has been challenged in recent years by studies reporting evidence of prenatal microbial colonization resulting from exposure to microbial communities of the placenta and amniotic fluid (6). In a carefully controlled study of 537 placentas, de Goffau et al. found no evidence for the existence of a placental microbiome (7). Further, a comprehensive review of the literature found that the existence of a fetal microbiome is not supported (8).

Within the first few hours of life, infants are exposed to multiple microbial microenvironments that may contribute to the infant's own microbiome, including those of the maternal gut, vagina, and skin. The mode of delivery represents a major early microbial exposure event that shapes the development of the infant microbiome, and therefore has implications for health in infancy and childhood.

The gut microbiome of vaginally delivered infants more closely resembles their mothers' vaginal microbiome, while newborns born by cesarean section have an increased prevalence of skin and environmental microbes (9, 10). Compared to vaginally born infants, intestinal colonization by *Bacteroides* spp. was impaired in infants born by cesarean section (9, 11–13). Vaginal delivery also favored colonization with members of the phylum Actinobacteria (9, 14, 15). Maternal dietary factors also influence the infant microbiome, although this effect varies by mode of delivery (16).

In contrast, the presence of opportunistic microorganisms was more prevalent in infants born by cesarean, including *Clostridium difficile* (12). Cesarean section delivery is also associated with reduced diversity of the infant gut microbiota over the first 3 months of life (17). By 6 months of age diversity within the 4 most common phyla (Bacteroidetes, Proteobacteria, Firmicutes, and Actinobacteria), did not vary by mode of delivery (17). The distortions observed in the infant microbiome associated with mode of delivery typically disappear in the first year of life (9, 11, 14, 18, 19).

### Mode of Feeding

Mode of feeding also has a significant influence on the development of the infant gut microbiome (20), which in turn differs in variety and abundance in infants who are formula fed compared to breastfed. The intestinal microbiome of the mother influences the development of the infant *via* breastfeeding (21) and an overlap is observed between the composition of the infant stool microbiome and that of both the maternal gut and breastmilk (22). Over the first month of life, a quarter of the bacteria in the stool of primarily breastfed infants was derived from breastmilk, the microbiome of which is dominated by members of the phylum Proteobacteria (1). Areolar skin, which is predominantly colonized by members of the phylum Firmicutes (1), is another source of microbial exposure distinguished by mode of feeding. Firmicutes is one of the two dominant phyla in a healthy adult (5), therefore exposure of infants to maternal areolar skin bacteria *via* breastfeeding affects the overall infant microbiome composition.

Breastfeeding is also associated with an increased abundance of probiotic organisms, including *Lactobacillus* and *Bifidobacterium* (9), the latter of which is typically the dominant organism in breast-fed infants (23). In contrast, formula feeding is associated with reductions in probiotic microbes including *Lactobacillus* and *Bifidobacterium* (3) as well as an expansion of *Bacteroides* (12, 24) and potentially enteropathogenic species including *Escherichia coli* and *C. difficile* (3, 12). Consequently, lack of breastfeeding has been associated with childhood health effects similar to those associated with cesarean deliveries.

Human milk oligosaccharides (HMO) and extracellular vehicles (EV) found in breast milk also influences the development of the infant microbiome. HMO are soluble complex carbohydrates which are indigestible to the infant. They function as a prebiotic, binding pathogenic bacteria, and modulating the intestinal immune response to promote beneficial bacteria (25), such as *Bifidobacterium*. While EVs carry mRNA, miRNA, cytosolic, and membrane proteins, and are implicated within cell-to-cell signaling further developing the infant microbiome (25).

### Environmental Factors

The definition of environmental effects on the infant microbiome covers a broad category that involves both intrauterine exposures (such as maternal disease, maternal diet, and maternal medications), and the postnatal environment (such as geography, and diet); these have been previously reviewed in references (26, 27). Although an important contributor to the development of the infant microbiome, environmental factors such as geography are less susceptible to manipulation.

Exposure to medications such as antibiotics, whether due to maternal use or post-natal treatment, is an area that can be clinically adjusted based on the potential to significantly disrupt the intestinal microbiota. Studies have shown that antibiotic usage during pregnancy disrupts the intestinal microbiota of infants in both mice (28, 29) and humans (30). Infant antibiotic treatment was associated with reduced quantity of *Bacteroides* and *Bifidobacteria* (5, 12), while the effects of maternal intrapartum antibiotic prophylaxis (IAP) were most pronounced in the reduction of Bacteroidetes, as measured at 3 months of age (19). The role of antibiotics in the intestinal microbiome is complex and multi-dimensional. Antibiotics may restore balance through the elimination of pathogenic microbes, but not without affecting commensal organisms.

### Genetic Influences

One of the earliest influences or determinants of what the microbiome looks like is attributable to the influence of host genetics. The relationship between genes and the human microbiome was recently reviewed (31–33). In brief, twin studies show that there are heritable taxa, most notably from the family Christensenellaceae (phylum Firmicutes), and heritability ranges from 0.1 to 0.3 (31) but can be as high as 0.67 based on sibling studies (34). Furthermore, certain genotypes or single nucleotide polymorphisms (SNP) may result in altered diet or altered immune system function. For example, the lactase gene (which confer lactose intolerance) is associated with abundance of *Bifidobacteria* and even more importantly, there is the interplay of genes associated with complex diseases like obesity and diabetes that play a role, although less so in the infant phase. Consequently, the interaction between genetics and other factors in the infant, including the immune system, results in a distinct composition of the microbiome.

## The Microbiome and the Immune System

The role of the intestinal microbiome in the modulation of immune responses is evidenced by the association between dysbiosis and immune-mediated diseases including diabetes (35, 36), Crohn's disease (37), rheumatoid arthritis (38), multiple sclerosis (39), inflammatory bowel disease (40), allergies (41), colorectal cancer (42), hypertension (43), and artherosclerosis (44). However, the mechanism(s) underlying these associations are still largely unknown, and much remains to be determined about how microbial dysbiosis during infancy impacts on the development of the immune system (29). In this review, we highlight how studies involving germ-free mice and probiotics administration have furthered our understanding of the interplay between the microbiome and immune development and/or function.

### Insights From Germ-Free Mice

Experiments with germ-free mice have demonstrated the importance of the gut microbiome in healthy immune development. Infant mice lacking a microbiome demonstrated reduced quantity and function of lymphoid cells (45). These findings are reflected in human studies. Colonization of the infant intestine with *Bacteroides fragilis* at 1 month of age was associated with increased maturation of IgA-secreting cells at 2 months of age (46), suggesting that the microbiome plays a role in the priming of the infant immune system (47).

The lack of a mature microbiome in infancy contributes to a bias toward a T-helper (Th) cell 2 response (48). In germ-free mice, the Th1 response is reduced and the Th1/Th2 balance is skewed toward a Th2 response (49). However, the Th1 response can be restored by a bacterial polysaccharide (PSA) associated with *Bacteroides fragilis* (50). Colonization of germ-free mice with segmented filamentous bacteria was associated with the induction of Th17 cells (51). Additionally, adaptive immunity to pathogens can be altered both directly and indirectly by interaction with specific commensal microbes (48, 52). These responses can include inducing the differentiation of IgA-producing B cells, as well as the expansion of Th17 and T-regulatory (Treg) cells (48, 52).

Furthermore, although the majority of studies conducted thus far have investigated the effect of the microbiome on the immune system, there is also evidence demonstrating that the immune system facilitates the maintenance of factors essential to the host-microbe relationship (53). The lack of TLR5 expression in the gut epithelium of neonatal mice influenced the composition of the intestinal microbiome including increased representation of Bacteroidetes and Clostridia taxa relative to wildtype (54). It has also been demonstrated that polymorphisms in both innate and adaptive immune pathways influence microbial composition in mice (55).

### Insights From Administration of Probiotics

Further mechanistic evidence may be derived from studies that have attempted to enhance immune responses through the administration of probiotics. Infants who received supplementation with *Lactobacillus acidophilus* for the first 6 months of life demonstrated reduced IL-10 responses to tetanus toxoid (TT) vaccine (56). Supplementation with *Lactobacillus acidophilus* for the first 6 months of life had no effect on innate immune responses (56); however, in response to polyclonal stimulation with SEB, production of IL-5 and TGF-beta increased in the probiotic group (56). In a group randomly assigned a 6-month course of probiotics, endotoxin-induced IL-6 levels were significantly reduced compared to controls, while no difference was detected in IFN-y production (57).

There is also evidence that *Bifidobacteria* has a role in the maturation of the infant immune system, and one way to alter the gut microbiome is *via* the addition to formula. Infants given formula supplemented with *Bifidobacterium longum* BB536 had increased *Bifidobacteria* and a higher *Bifidobacteria*/*Enterobacteriaceae* ratio at 2 and 4 months of age, as well as a higher number of IFN-γ secreting cells and an increased ratio of IFN-γ/IL-4 secreting cells at 7 months of age, indicating that BB536 may contribute to the bias toward a pro-Th1 response (58, 59). Although outside the scope of this review, probiotics use in the treatment of antibiotic-associated diarrhea in pediatrics has some utility (59, 60); however, results are strain specific and evidence is lacking. There are also risks that should be considered such as the potential that translocation of bacteria in probiotics may cause adverse health effects.

Finally, there is significant inter-individual variability (61) of the microbiome in humans. Together with genetic diversity, this presents a challenge in the investigation of host–microbe interactions (62). Therefore, the clinical utility of probiotic supplementation, especially in younger individuals, requires additional studies targeting risks and benefits.

## The Microbiome and Vaccines

Given the associations between the microbiome and the function and composition of the immune system, the potential implications for vaccine responsiveness have been an area of interest. There is evidence implicating the host intestinal microbiome in the development of vaccine-specific immunity, although the mechanisms by which the microbiome modulates vaccine immune response are largely undetermined.

In addition to influencing microbial composition, the role of TLR5 in the microbiome also influences vaccine responses. A study in mice showed that TLR5-mediated sensing of flagellin that was produced by the gut microbiota was necessary for antibody responses to the influenza vaccine, as mice deficient for TLR5 had substantially impaired responses to the vaccine at day 7 and 14 post-vaccination (63), while germ-free (GF) or antibiotic-treated mice had significantly impaired antibody responses to the influenza vaccine (62–64). These data suggest that the gut microbiota enhances systemic vaccine responses but may suppress oral vaccine responses (65).

While several studies have reported an association between the composition of the infant intestinal microbiome and the quality of their response to vaccines (Table 1), the findings to date have been inconsistent. Furthermore, studies aimed at the effect of intramuscular or subcutaneous vaccines on the microbiome are still lacking.

**Table 1:** Association between microbiome and vaccine response in infants (up to age 2).

**Vaccine**	**Population**	**Effect/Association**	**Citation**
Rotavirus	Pakistani infants age 6 weeks	Increased levels of Firmicutes and Proteobacteria in responders. Increased *E. coli* and *Serratia* in non-responders	(48)
Rotavirus	Ghanaian infants age 6 weeks	Abundance of phylum Bacilli positively correlated with RV response, phylum Bacteroidetes correlated with RV non-response	(66)
OPV, BCG, TT, HBV	Bangladeshi infants age 15 weeks	Relative abundance of Actinobacteria was positively associated with T-cell proliferative responses to OPV, BCG and TT, and to increased OPV and TT-specific IgG responses at 15 weeks of age.	(67)
OPV, BCG, TT, HBV	Bangladeshi infants aged 15 weeks to 2 years	*Bifidobacterium* abundance positively associated with CD4 T-cell response to BCG, TT, and HBV, at 15 weeks and to BCG and TT at 2 years, and with TT-specific plasma IgG and OPV-specific stool IgA at 2 years	(68)
Rotavirus	Indian infants age 6 and 10 weeks	No significant difference in intestinal microbial diversity between infants who seroconverted to RV vaccine and those who did not	(69)
Rotavirus	Nicaraguan infants age 8 weeks to 6 months	No statistically significant difference in intestinal microbiome composition between infants that responded to vaccine and those who did not	(70)

*OPV, oral polio vaccine; BCG, Bacillus Calmette-Guerin; TT, tetanus toxoid; HBV, hepatitis B vaccine; OTV, oral typhoid vaccine*.

### Effects of Dysbiosis on Vaccine Responses

The infant immune response is skewed toward a Th2 response dominated by IL-4, IL-5, and IL-10 cytokine production (71) and Th2 cells are associated with the development of allergies (72). Because the infant intestinal microbiome is uniquely susceptible to disruption, deficits or disruptions in microbial communities that biases toward a Th2-type response (48, 49, 73) could be related to the association between intestinal dysbiosis and allergy development (41). On the other hand, colonization with certain bacteria (i.e., *Bifidobacteria*) can shift the balance toward a Th1 response (58).

Dysbiosis may also be induced *via* antibiotics as shown in both human and mice studies. Patterns of bacterial colonization due to gestational age and may be in part due to early antibiotic exposure in the premature infant secondary to treatment for infections (i.e., sepsis, necrotizing enterocolitis) (74). Antibiotic-induced dysbiosis in early-life resulted in reduced specific IgG responses to five different vaccines, although following fecal transfer of commensal microbiota, immunity was restored (28). Additionally, infant gut dysbiosis induced by maternal antibiotic usage was associated with reduced adaptive antiviral immunity in response to infection with Vaccinia-OVA (29). In contrast, antibiotic administration did not impaired vaccine responses in adult mice (28) and did not influence anti-rotavirus (RV) IgA titres in adult humans (52). These findings support the hypothesis that in cases of dysbiosis, vaccine responses can be boosted through manipulation of the microbiome in infants (not necessarily in adults), although details such as the duration of therapy required to adequately restore disruptions to the infant microbiome still need to be determined.

Finally, impaired vaccine responsiveness has been reported in low-income settings compared to wealthier regions (75–77). These regions often experience conditions of reduced sanitation and a correspondingly high burden of enteric pathogens (3), which may result in increased dysbiosis. It has been suggested that this excessive pathogenic burden could contribute to the reduced efficacy of oral vaccines that is often observed in the developing world (78, 79). Evidence from a Zimbabwean cohort supports this hypothesis, as improvements in sanitation were associated with improved responsiveness to the rotavirus (RV) vaccine (80).

### Effects of Probiotics on Vaccine Responses

Given that the intestinal microbiome of infants is still developing and is characterized by low diversity and rapid change (27), it has been surmised that the infant microbiome may be much more susceptible to influence from external factors such as probiotic supplementation (3). While probiotics have produced beneficial effects in the treatment of some autoimmune conditions (81, 82), studies have also investigated the use of probiotics and prebiotics as adjuvants to enhance vaccine responses, the results of which have been thoroughly reviewed elsewhere (3, 83). Thus far, the results have been highly inconsistent in infant populations, with variations between studies in the type, dose, and duration of probiotic supplementation further complicating any conclusions that may be drawn from them regarding the utility of probiotics as vaccine adjuvants.

At 4 months of age, infants given enhanced formula to promote growth of intestinal *Bifidobacteria* displayed an enhanced polio-specific response following vaccination and a positive correlation was observed between total *Bifidobacteria* as a percentage of intestinal flora and titres of anti-poliovirus IgA (47). Infants given *Lactobacillus rhamnosus* GG (LGG) had an increased rate of rotavirus IgA seroconversion (84), while infants given a cereal supplemented with *Lactobacillus paracasei* F19 (LF19), from 4 to 13 months of age, demonstrated an increase in immune responses to diphtheria following vaccination, with no difference in Hib or tetanus-specific IgG levels (85). A combination of four probiotic strains was associated with increased seroconversion rates in response to vaccination with Hib, but failed to elicit a significantly enhanced response to either tetanus or diphtheria (86). Additionally, probiotic supplementation appeared to positively influence anti-HBsAg responses when the vaccine series included two monovalent vaccines and concluded with a quadrivalent vaccine at 6 months, although this trend did not reach statistical significance; however, no effect was observed when a 3-dose monovalent series of HBV was administered (87).

In contrast, a few studies have even reported a negative association between probiotics and vaccine responses. Maternal supplementation with *Lactobacillus rhamnosus* GG (LGG) was associated with a reduction in infant antibody responses to tetanus, Hib, and pneumococcus vaccines (88), and Bangladeshi children who received a 4-week course of BBG-01 had reduced levels of cholera toxin subunit B-specific IgA levels compared to controls (89). The administration of probiotics was associated with an increase in secretory IgA in stool samples of children at 5 months of age (90); however, this did not translate to improved clinical outcomes, as infants exposed to probiotics reported a higher frequency of mucosal infections up to 2 years of age, despite both groups being able to clear infections at comparable rates (90).

Just as the bacterial composition of the intestine can influence the response to oral vaccines, it has also been proposed that the administration of oral vaccines can cause a perturbation that would be reflected in alterations to the microbiome following vaccination (91). However, few studies have investigated this possibility to date. These conflicting trends confirm that a more thorough understanding of the interaction between the intestinal microbiome and the development of vaccine-specific immunity is necessary before probiotic supplementation can be developed as an effective adjuvant to improve vaccine responses.

## Methodologies for Analysis of the Microbiome

While the most commonly analyzed samples are derived from stool, tissue biopsies allow for actual visualization relative to tissue structure as well as evaluation of adherence microbes. Methods used to study the human microbiome have been previously described (92–94) and involve using DNA-based methods (metagenomics), RNA-based approaches (metatranscriptomics), protein-based approaches (metaproteomics), and metabolite-based approaches (metabolomics).

## Conclusion and Future Research Directions

The infant microbiome is a rapidly evolving and dynamic environment that is determined and influenced by a variety of internal and external factors. This dynamic microbial environment is integral in modulating the infant's immune function and composition, which in turn has implications for vaccine specific responsiveness. A great deal more research is required to establish a definitive connection between microbial composition and vaccine responsiveness. From the limited data available, the interaction between microbiome and vaccine response varies by vaccine and may also be influenced by other factors known to influence both vaccine responsiveness and microbial composition including age, gender, and route of administration.

Additionally, it has been hypothesized that the composition of the intestinal microbiome as it relates to the development of vaccine-specific immunity may be modulated by probiotic supplementation. Direct modulation of the intestinal microbiome would appear to favor the role of probiotics in influencing the response to oral vaccines; however, the emerging role of the microbiome in the function of systemic immunity suggests probiotics may benefit the response to parenteral vaccines as well. Furthermore, each individual vaccine has the potential to modulate the microbiome such that the vaccination schedule of each country influences the resulting microbiome due to bidirectional interactions between microbiome and vaccines.

Consequently, a better understanding of the factors that enhance or impede the immunological, mucosal, and microbial protection is required. There is a further need to understand the microbiome-immune interplay, and how one influences the other. To accomplish this, we recommend (1) longitudinal prospective cohort studies in infants across a broad geographical and socioeconomic spectrum to define the ontogeny of the microbiome ontogeny, (2) utilization of both *in-vivo, ex-vivo* and *in-vitro* methods to better understand how alterations in the microbiome affects the microbiome-immune interplay (either due to intentional manipulation *via* administration of an agent such as a probiotic or dysbiosis secondary to disease), (3) a standardized approach to evaluate the microbiome in order to allow comparisons among different cohorts (i.e., 16S rRNA, metagenomics, metatranscriptomics, and metabolomics) (94, 95), and (4) a systematic integration of data from various molecular approaches to define the dominant microbial profile at different stages of life and how they change over time.

We believe the above strategies will not only improve the gaps in understanding the microbiome-immune system-vaccine interplay but could also help decrease mortality and morbidity in the pediatric population by developing potential therapeutic interventions and enhancing immunogenicity.

## Author Contributions

CR, OO, and KS conceived the manuscript, co-wrote and reviewed the content. All authors contributed to the article and approved the submitted version.

## Conflict of Interest

The authors declare that the research was conducted in the absence of any commercial or financial relationships that could be construed as a potential conflict of interest.

## Abbreviations

BCG: Bacillus Calmette-Guerin
DNA: deoxyribonucleic acid
HBV: hepatitis B vaccine
HBsAg: hepatitis B surface antigen
Hib: *Haemophilus influenzae* type B vaccine
IFN: interferon
Ig: immunoglobulin
IL: interleukin
OPV: oral polio vaccine
OTV: oral typhoid vaccine
RNA: ribonucleic acid
RV: rotavirus vaccine
SEB: staphylococcal enterotoxin B
Th: T-helper
TLR: Toll-like receptor
and TT: tetanus toxoid.
